# Functions of the Essential Gene *mraY* in Cellular Morphogenesis and Development of the Filamentous Cyanobacterium *Anabaena* PCC 7120

**DOI:** 10.3389/fmicb.2021.765878

**Published:** 2021-10-21

**Authors:** Jing Liu, Wei-Yue Xing, Ju-Yuan Zhang, Xiaoli Zeng, Yiling Yang, Cheng-Cai Zhang

**Affiliations:** ^1^State Key Laboratory of Freshwater Ecology and Biotechnology, Institute of Hydrobiology, Chinese Academy of Sciences, Wuhan, China; ^2^College of Advanced Agricultural Sciences, University of Chinese Academy of Sciences, Beijing, China; ^3^Institut WUT-AMU, Aix-Marseille University and Wuhan University of Technology, Wuhan, China

**Keywords:** peptidoglycan, lipid I, cell division, heterocysts, cyanobacteria, divisome, elongasome

## Abstract

Bacterial cell shape is determined by the peptidoglycan (PG) layer. The cyanobacterium *Anabaena* sp. PCC 7120 (*Anabaena*) is a filamentous strain with ovoid-shaped cells connected together with incomplete cell constriction. When deprived of combined nitrogen in the growth medium, about 5–10% of the cells differentiate into heterocysts, cells devoted to nitrogen fixation. It has been shown that PG synthesis is modulated during heterocyst development and some penicillin-binding proteins (PBPs) participating in PG synthesis are required for heterocyst morphogenesis or functioning. *Anabaena* has multiple PBPs with functional redundancy. In this study, in order to examine the function of PG synthesis and its relationship with heterocyst development, we created a conditional mutant of *mraY*, a gene necessary for the synthesis of the PG precursor, lipid I. We show that *mraY* is required for cell and filament integrity. Furthermore, when *mraY* expression was being limited, persistent septal PG synthetic activity was observed, resulting in increase in cell width. Under non-permissive conditions, filaments and cells were rapidly lysed, and no sign of heterocyst development within the time window allowed was detected after nitrogen starvation. When *mraY* expression was being limited, a high percentage of heterocyst doublets were found. These doublets are formed likely as a consequence of delayed cell division and persistent septal PG synthesis. MraY interacts with components of both the elongasome and the divisome, in particular those directly involved in PG synthesis, including HetF, which is required for both cell division and heterocyst formation.

## Introduction

Bacterial cell shape is determined primarily by the peptidoglycan (PG) sacculus. Peptidoglycan, or murein, is a giant, cell-sized macromolecule and an essential protective layer of bacterial cell envelope by providing mechanistic stability and reduced permeability (Bouhss et al., 2008). PG is composed of alternating units of *N*-acetylmuramoyl-peptides (MurNAc-peptides) and N-acetylglucosamine (GlcNAc), crosslinked together by peptide chains. In *E. coli*, the biosynthesis of PG is a complex process with multiple steps taking place from the cytoplasm to the inner membrane, till the periplasm where insertion of new subunits into the PG network is carried out by a FtsZ-mediated treadmilling mechanism (Addinall and Lutkenhaus, 1996; Hale and de Boer, 1999; Typas et al., 2012; Yang et al., 2017; Egan et al., 2020). At the inner membrane, one of the key enzymes is MraY (the phospho-MurNAc-pentapeptide translocase), a membrane enzyme responsible for the synthesis of lipid I, a precursor of PG biosynthesis (Ikeda et al., 1991). Lipid I is transformed into lipid II by MurJ, flipped into the periplasm and further processed into MurNAc-pentapeptide-GlcNAc (Egan et al., 2020). The latter is inserted into the PG layer as a building unit by various penicillin-binding proteins (PBPs) (Sauvage et al., 2008). MraY is a well-studied target for antibiotics and the bacteriophage ϕX174 for host cell lysis in *E. coli* (Bernhardt et al., 2000; Bugg and Kerr, 2019). Because of its rigid nature, PG synthesis and degradation occur constantly during cell growth and division for the morphogenesis of the newly formed cell poles during cell constriction. The spatiotemporary synthesis of the PG layer in a cell is directed by two cytoskeleton proteins, MreB and FtsZ, with each of them coordinating a large protein complex extending from the cytoplasm, across the cytoplasmic membrane, to the periplasm (Pinho et al., 2013). MreB-associated protein complex is called elongasome which promotes lateral insertion of PG along the cell wall. It is responsible for cell elongation during cell growth. The spherical bacteria usually lacks MreB. FtsZ, a tubulin homolog, is a central component of the divisome complex. FtsZ coordinates PG synthesis during cell division and is thus responsible for morphogenesis of the new cell poles during cell constriction. In bacteria lacking MreB, cell elongation is achieved by PG insertion at the division site in a FtsZ-dependent manner (Pinho et al., 2013). Specific to cyanobacteria, ZipN is a principal FtsZ tether to the membrane and an essential organizer of the divisome (Camargo et al., 2019).

Although cyanobacteria are Gram negative prokaryotes, they have a thick and multi-layered PG with extensive crosslinking in the periplasmic space, a characteristic resembling more Gram-positive bacteria. Cyanobacteria exhibit high diversity in cell shape, size and structure. Accordingly, their mode of PG synthesis shows a strong variation according to the strains examined (Zhang et al., 2018). In cyanobacteria such as *Anabaena*/*Nostoc* PCC 7120 (hereafter *Anabaena*), PG synthesis is also subject to developmental control. *Anabaena* is a filamentous freshwater cyanobacterium which is extensively used as a model for studying prokaryotic development and cell-cell communication. *Anabaena* can take up combined nitrogen sources such as ammonium or nitrate for its growth. Once deprived of combined nitrogen, about 5–10% of cells along the filaments differentiate within 20–24 h into heterocysts, cells specialized in atmospheric N_2_ fixation (Wolk et al., 1994; Zhang et al., 2006; Herrero et al., 2016). Heterocysts provide a micro-oxic environment so that the oxygen-labile nitrogenase can be functional. Under such conditions, vegetative cells perform oxygen-evolving photosynthesis and provide carbon source and reducing power to heterocysts, while receiving fixed nitrogen from the latter. Heterocysts have a distinct morphology with two extra layers deposited outside the membranes: an inner glycolipid layer as a barrier of oxygen diffusion and an outer layer of polysaccharides (Shvarev et al., 2018). In addition, heterocysts have PG layers that become thickened at the late steps of heterocyst differentiation. While *Anabaena* filaments share a common outer membrane with a continuous periplasm, each cell has its own PG layers and inner membrane. For some cells, the PG layers may be chemically connected since murein sacculus of several cells could be isolated together (Lehner et al., 2011). Nanopores are drilled through the polar PG layers by the action of amidases, in order to establish the Intercellular communicating channels made of proteins, known as septal junctions, through which carbon and nitrogen compounds can be exchanged along the filaments (Nürnberg et al., 2015; Bornikoel et al., 2017; Kieninger et al., 2019).

*Anabaena* has a large family of PBPs, and PBP2 (PBP1C, Alr5101) encoded by *pbpB* (Hahn and Schleiff, 2014) is required for diazotrophic growth (Lázaro et al., 2001). The *pbpB* mutant did not show a particular phenotype when cultured in the presence of a combined nitrogen, but displayed irregular cell size, cell shape, and shortened filament length (Lázaro et al., 2001). Another gene encoding a second class B PBP, *alr5045*, was also required for diazotrophic growth, as the corresponding mutant grew less well under such conditions (Burnat et al., 2014). FtsI (PBP3, Alr0718) acts at the cell division sites for cell constriction during cell division, it was unable to be inactivated completely (Burnat et al., 2014). Treatment with aztreonam that targeting to FtsI led to cell division defect, and affected heterocyst differentiation (Sakr et al., 2006). FtsI interacts with HetF, a protein required for heterocyst differentiation and participates in PG constriction during cell division under high light (Xing et al., 2021). Another developmental regulator PatD controls the FtsZ activity in developing cells, and may be responsible for the control of heterocyst cell size with increased PG synthesis (Wang et al., 2021). These studies indicate that the PG layers are important for heterocyst development and functioning. However, since a large family of PBPs exist in *Anabaena*, with some being essential, and others likely redundant, it is therefore difficult to pinpoint the role of PG metabolic pathway in *Anabaena*.

In this study, we took a different approach to study the function of PG in this organism, by focusing on the role of *mraY* gene that is required for the biosynthesis of the PG precursor, lipid I. *Anabaena* has only one copy of *mraY* gene, and we found that MraY could interact with several components of the divisome such as FtsQ and ZipN, as well as PBP1A and HetF. By analyzing the function of *mraY* in *Anabaena* using a conditional mutant, we demonstrate that MraY is essential for cell shape, cell and filament integrity, as well as heterocyst differentiation. These results show that the integrity of the PG layers is essential for both vegetative growth and heterocyst formation.

## Results

### *mraY* Is Essential in *Anabaena*

**FIGURE 1:**
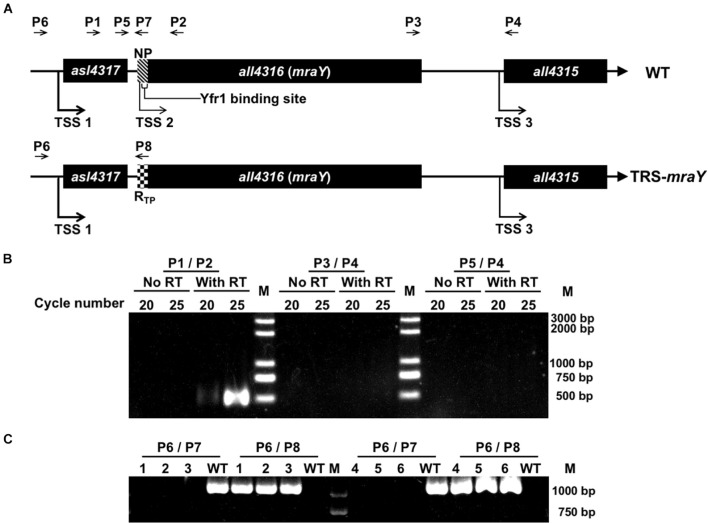
Construction of the conditional mutant of *mraY* using CRISPR-Cpf1. **(A)** The schematic representation of the genotype of wild-type *Anabaena* (WT) and the TRS-*mraY* strain, and their surrounding genetic context. NP, native ribosome binding site (–39 to –1 bp) of *mraY*. R_*TP*_, theophylline riboswitch. Yfr1 binding site, at –23 to –6 bp of *mraY*, is also shown. The thickness of the arrow represents the relative strength of the transcription starting site (TSS), based on published RNA seq data (Mitschke et al., 2011). The relative position of different DNA primers (P1–P8) used are shown. **(B)** RT-PCR for the *mraY* gene cluster. P1, P2, P3, P4, and P5 (see **A** for their relative position) correspond to oligonucleotides Pall4316F280m, Pall4316R220, Pall4315F452m, Pall4315R48, and Pall4315F1702m, respectively. The expected size of the RT-PCR product amplified from the WT genome with P1 and P2 or P3 and P4, is 500 bp, respectively, and that amplified with P5 and P4 is 1,750 bp. 20 and 25 correspond to the number of PCR cycles used for amplification. **(C)** Verification on the genotype of TRS-*mraY* (clones 1-3) and *ftsZ-cfp*:TRS-*mraY* (clones 4–5) by PCR using the primers as shown in **(A)**. P6, P7, and P8 are the oligonucleotides Pall4316F1047m, Pall4316R1m, and PV-19, respectively. Three independent colonies grown up on the conjugation plates were examined. The expected size of the PCR product amplified from the WT genome with P6 and P7 is 1,047 bp, and with P6 and P8 is 0 bp. The expected size of the PCR product amplified from TRS-*mraY* mutant with P6 and P7 is 1,071 bp, and no product should be amplified with P6 and P8.

In many bacteria, *mraY* is found in the gene cluster with other *mur* genes and several genes involved in cell division (Boyle and Donachie, 1998; Mohammadi et al., 2007; Egan et al., 2020). In *Anabaena*, *mraY* (*all4316*) is surrounded by genes mostly encoding proteins of unknown function. The gene cluster includes 3 open-reading frames (ORFs) (Figure 1A). *asl4317* and *all4315* are found on the two sides of *mraY*, and all transcribed in the same direction (Figure 1A). The distance between the ORF of *asl4317* and that of *mraY* is 78 bp, while the ORF of *mraY* and that of *all4315* is separated by 404 bp.

According to the RNA seq data (Mitschke et al., 2011), each of the three genes possesses an independent promoter. To check if they could be cotranscribed, RT-PCR was performed, using oligonucleotide primers covering the intergenic regions. As shown in Figure 1B, co-transcript of *asl4317* and *mraY* was identified by RT-PCR using primers P1/P2, but no signal was detected with primers P3/P4 (covering *mraY* and *all4315)* or P4/P5 (covering all three genes). This result suggests that *mraY* is cotranscribed with *asl4317*, but not with *all4315*. The RNA-seq data suggests that *mraY* may have a 10-times weaker promoter of its own compared with the shared one in front of *asl4317* (Mitschke et al., 2011).

In order to make genetic analysis on the function of *mraY*, we initially tried to inactivate *mraY* via in-frame markerless deletion. However, no colonies were obtained on plate after conjugation, suggesting an essential function of *mraY*, consistent with previous reports of *mraY* in other bacteria (Boyle and Donachie, 1998; Bouhss et al., 1999; Pearcy et al., 2021). Using Cpf1-based gene editing technique (Niu et al., 2018), we created a conditional mutant of *mraY* (TRS-*mraY*) by replacing its weak and native promoter with a tunable synthetic riboswitch, R_*TP*_, induced by theophylline (TP) (Nakahira et al., 2013; Figure 1A). When replaced by R_*TP*_, the region corresponding to the binding site of the small RNA Yfr1, a negative regulator (Brenes-Álvarez et al., 2020), was deleted at the same time, making the expression of *mraY* only dependent on the translational control by R_*TP*_. Translation of the *mraY* mRNA occurs normally in the presence of TP, but this process was blocked by the riboswitch when TP is removed. The obtained TRS-*mraY* mutant was first checked for gene segregation using specific primer targeting either the native promoter of *mraY* or R_*TP*_. As shown in Figure 1C, for all three independent clones of strain TRS-*mraY* mutant, no WT copy could be detected using primers P6/P7, while a DNA fragment corresponding to the replaced promoter region could be amplified using primers P6/P8. These results indicate that mutants were fully segregated.

**FIGURE 2:**
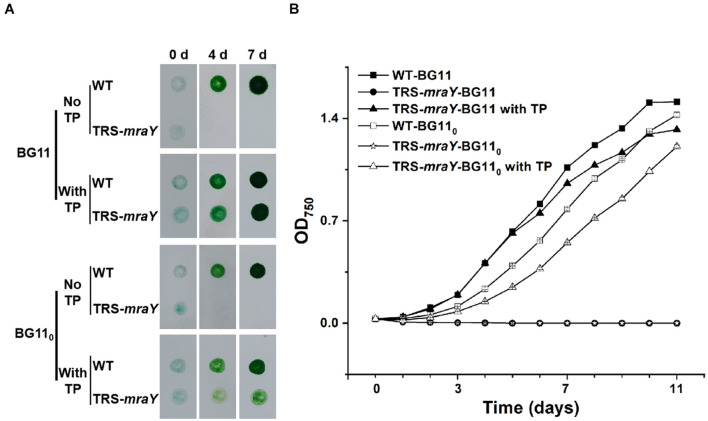
The growth of TRS-*mraY* in the BG11 and BG11_0_ media, under permissive and non-permissive conditions. **(A)** *Anabaena* WT and TRS-*mraY* in the BG11 and BG11_0_ on agar plates supplemented with 1 mM of theophylline (With TP) or without TP (No TP). Similar volume of precultures of TRS-*mraY* or WT at OD_750_ 0.5 was spotted on agar plates, which were photographed after the indicated time (in days) of incubation. **(B)** Growth curves of TRS-*mraY* and WT in BG11 and BG11_0_. Absorbance at 750 nm was measured daily as indicated following inoculation in the BG11 and BG11_0_ liquid media supplemented with 1 mM of TP or without TP.

To test the growth capacity, TRS-*mraY* was first grown in the presence of 1 mM of TP in BG11 culture medium (with nitrate as nitrogen source), then equal culture volumes with comparable optical density were concentrated and spotted on BG11 or BG11_0_ (deprived of combined nitrogen) plates with or without TP. After 4 and 7 days of incubation, the mutant spots were completely bleached compared with WT spots in the absence of TP, while cells maintained in the presence of 1 mM of TP were able to grow as indicated by the green color (Figure 2A). The mutant was also tested in liquid culture in flasks in both BG11 and BG11_0_ (Figure 2B and Supplementary Figure 1). Similar result was observed that in the presence of 1 mM of TP, mutant cells were able to grow although slightly slower than the WT control, but cells without TP addition in the medium died completely (Figure 2B and Supplementary Figure 1). These results demonstrate that *mraY* is essential in *Anabaena*. The TRS-*mraY* strain could be maintained in the presence of 1 mM of TP as permissive conditions, and its phenotype could be analyzed upon transferring to non-permissive conditions by removal of TP from the growth medium.

**FIGURE 3:**
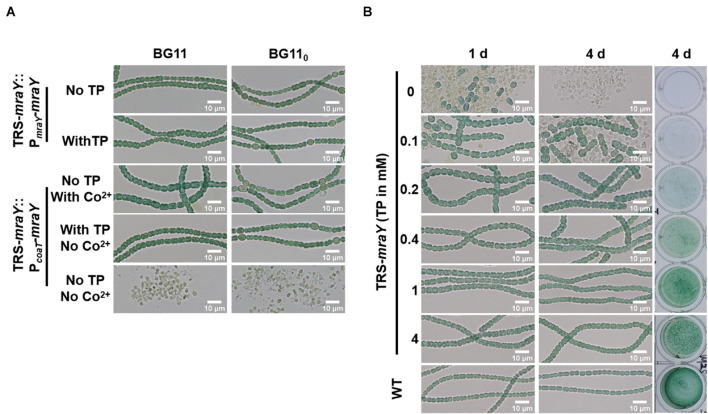
The morphology of cells and filaments. Samples of the cell suspensions from fresh cultures, obtained under different conditions as indicated, were directly observed under a light microscope and photographed. The size in indicated by a bar. **(A)** TRS-*mraY* mutant complemented with pP*_*mraY*_-mraY* or pP***_*coaT*_***-*mraY* grown in liquid BG11 or BG11_0_ medium. P*_*mraY*_* is promoter region in front of TSS 1 sequence depicted in Figure 1A. P*_*coaT*_* is a Co^2+^ or Zn^2+^ inducible promoter cloned from *Synechocystis* sp. PCC 6803. See text for more details. **(B)** Filaments of strain TRS-*mraY* grown in BG11 medium with 1 mM of TP were collected and transferred to BG11 medium without TP for 12 h to deplete residual TP (Supplementary Figure 5). Then filaments were transferred to BG11 medium with different concentrations of TP, incubated for 1, or 4 days, observed by light microscope. The same liquid cultures in vials at 4 days were also photographed (right panel). WT was used as the control.

To exclude any polar effect in TRS-*mraY* due to the replacement of the promoter region and further ascertain the essential function of *mraY*, complementation assays were carried out with two different plasmid constructs (Figure 3A). The first one, pP*_*mraY*_-mraY*, carried *mraY* coding region placed behind the native strong promoter TSS 1 as depicted in Figure 1A; the second plasmid, pP*_*coaT*_-mraY*, carried the coding region of *mraY* with a cobalt-inducible promoter of *coaT* (Peca et al., 2008). The strain carrying pP*_*mraY*_-mraY* grows normally with or without TP in both BG11 and BG11_0_. However, the strain with pP*_*coaT*_-mraY* showed extensive cell lysis in the absence of any inducers (TP and Co^2+^), and this defect was corrected upon addition of TP that allows the translation of *mraY* from the chromosome or Co^2+^ that allows the expression of *mraY* from the replicative plasmid. In both complemented strains, heterocyst development could be seen, consistent with their growth under diazotrophic conditions in BG11_0_ (Figure 3A).

### MraY Is Required for Maintaining Cell and Filament Integrity and Cell Shape

To dissect the essential functions of *mraY*, we analyzed the morphology of cells and filaments of TRS-*mraY* under permissive and non-permissive conditions (Figure 3B). After 4 days of culture, the viability of TRS-*mraY* increased as the concentration of the inducer increased. With 1 or 4 mM of TP, the mutant grew almost as the WT, consistent with the growth curves as shown in Figure 2B. Without TP, or just with 0.1 mM of TP, extensive cell lysis, and filament fragmentation was found. In the presence of low concentrations of inducers, cell shape appeared to be different from that of the WT (Figure 3B).

**FIGURE 4:**
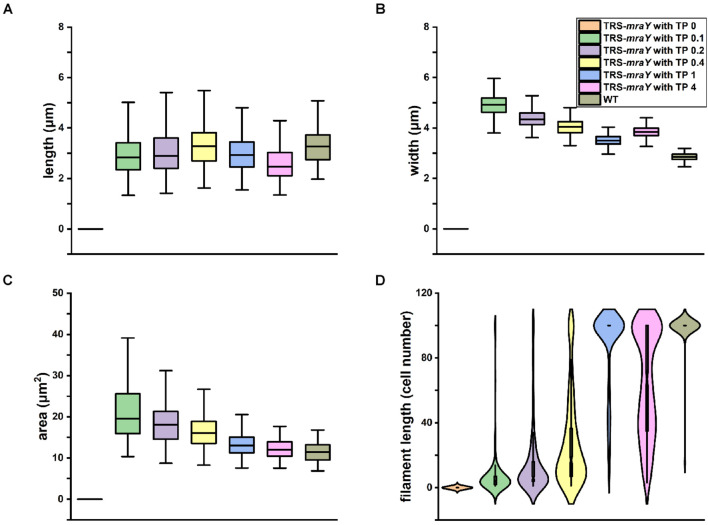
Effects of *mraY* in filament integrity and cell morphology. WT and TRS-*mraY* mutant incubated for 4 days with different concentrations of TP were analyzed for cell length **(A)**, cell width **(B)**, cell area or volume **(C)**, and filament length **(D)**. 400 cells were measured for cell length and width, 300 cells for cell area, and 300 filaments were analyzed for filament length. In the latter case, filaments with 100 cells include those with more than 100 cells per filament.

We quantified cell shape changes as well as filament integrity in the WT and the TRS-*mraY* mutant in the presence of different concentrations of TP after 4 days of incubation. As shown in Figure 4, cell length based on analysis of 400 cells, displayed little changes with or without TP. However, cell width gradually increased as the concentrations of the inducers increased. In WT, the average width of the cells is about 2.84 μm. But with 0.1 mM of TP, the width of TRS-*mraY* increases to 4.92 μm. These changes translated into increased cell area in the conditional mutant incubated with low concentrations of TP as compared to the same strain with TP, or the WT (Figure 4C), which is also consistent with the microscopic images as shown in Figure 3. Consequently, under non-permissive conditions, cell shape of the mutant depleted of MraY became more rounded in shape compared to the ovoidal shape in WT.

Quantitative analysis demonstrates that filament integrity is strongly affected by the expression levels of MraY (Figure 4D and Supplementary Figure 2). While under non-permissive conditions, cells were completely lysed after 4 days of incubation, beginning with 0.1 mM of TP, some cells could survive after 4 days of culture. When either 0.2 or 0.4 mM of TP was added to induce the expression of *mraY* in the TRS-*mraY* mutant, some long filaments were found, but most of the filaments were still short with less than 50 cells. Compared to the WT in which most of the filaments had more than 100 cells per filament, TRS-*mraY* mutant had the majority of filaments with more than 100 cells per filament only when 1 mM of TP was added to the culture, indicating that this is the most suitable conditions to restore the phenotype of the TRS-*mraY* mutant. In the presence of 4 mM of TP, the TRS-*mraY* mutant had 35% of the filaments with more than 100 cells but also shorter filaments too, which may be resulted from overexpression of *mraY*. Thus, down regulation of *mraY* expression has a strong impact on cell shape, cell integrity and filament length.

### Persistent Septal Peptidoglycan Synthesis After Downregulation of *mraY* Expression

**FIGURE 5:**
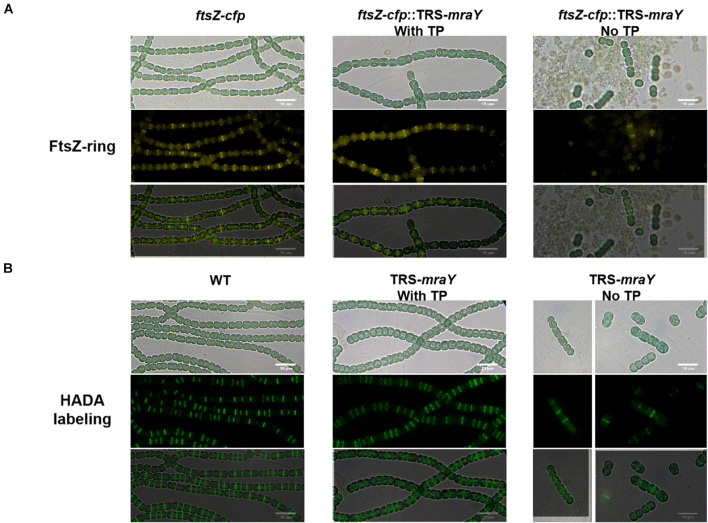
FtsZ-ring formation and PG synthesis in TRS-*mraY* in BG11 under permissive and non-permissive conditions. **(A)** FtsZ-CFP is a CFP fluorescence fusion protein of FtsZ as already reported (Xing et al., 2021). Filaments of strain *ftsZ-cfp*:TRS-*mraY* grown in BG11 medium with 1 mM of TP were collected and transferred to BG11 medium without TP for 12 h (Supplementary Figure 5) to remove TP. Then they were incubated for 24 h in BG11 without TP to observe FtsZ localization. **(B)** Strain TRS-*mraY* was first treated similarly to remove TP (Supplementary Figure 5), then incubated for 24 h with 150 μM of HADA in BG11 without TP to observe PG synthesis pattern. WT was used as the control.

Bacterial cell shape relies on PG synthesis under the control of the two protein complexes, the elongasome directing lateral PG synthesis along the side wall contributing to cell length, and the divisome for PG synthesis at the division site responsible for cell width (Szwedziak and Löwe, 2013). The increase in cell width of the TRS-*mraY* mutant under non-permissive conditions suggests enhanced PG synthesis activity at the division site. Therefore, we examined whether FtsZ-ring formation and PG biogenesis are altered in the TRS-*mraY* mutant under permissive and non-permissive conditions. For this, we constructed a *ftsZ-cfp*:TRS-*mraY* strain in which chromosomal copy of *ftsZ* was replaced by *ftsZ-cfp* fusion (Wang et al., 2021). The fluorescence of FtsZ-CFP (cyan fluorescent protein) was then observed. Under permissive condition (in the presence of 1 mM of TP), FtsZ-ring formation was similar to WT (Figure 5A). At 24 h after the removal of TP from the growth medium, extensive filament fragmentation and cell lysis occurred as already shown in Figure 3. Nevertheless, FtsZ-CFP localization could be found in short filaments, indicating that just before cell lysis, FtsZ was still targeted to midcell (Figure 5A). To visualize PG biosynthesis, we used HADA (7-hydroxycoumarin-amino-D-alanine), a fluorescent analog of the PG precursor D-Ala that can be incorporated into live cells through the transpeptidase activity (Hsu et al., 2017; Zhang et al., 2018). Under non-permissive conditions incubated for 24 h, we also observed the localization of HADA incorporation at midcell and cell-cell junctions (Figure 5B). In comparison to the WT control, most of the septa in the TRS-*mraY* strain under different conditions were wider, suggesting that cell division was not completed properly (Figure 5 and Supplementary Figure 3).

To analyze the localization pattern based on the images as shown in Figure 5, we quantified the percentage of cells that have FtsZ-CFP or HADA staining, either at midcell corresponding to active PG remodeling at the division site, or at cell-cell junctions reflecting PG precursor incorporation from the precedent cell cycle (Table 1 and Supplementary Table 4). In both WT and TRS-*mraY* mutant incubated in the presence of 1 mM of TP, 53.6 and 52.6% of the cells displayed FtsZ-CFP at midcell. However, 1 day after the removal of TP, only 30% of the cells showed FtsZ-CFP at midcell, indicating less cells initiating cell division without the induction of *mraY*, possibly due to the lethal effect of downregulation of *mraY*. In contrast, 44% of the cells retained FtsZ-CFP at cell-cell junctions, as compared to 23.7% of the cells of the same strain incubated with 1 mM of TP, a percentage similar to that of WT. Therefore, downregulation of *mraY* decreased initiation of cell division as indicated by the localization of FtsZ-CFP, but more FtsZ-CFP persisted at the cell-cell junctions than the controls (WT and mutant with TP), suggesting that the FtsZ targeted to the division sites at the precedent cycle was less likely to be disassembled at the cell-cell junctions in comparison to the control. Interestingly, even a concentration as low as 0.1 mM of the TP inducer could restore the capacity of the mutant in FtsZ-CFP localization at the midcell; however, persistent FtsZ-CFP localization at the cell-cell junction could be complemented only by the addition of 1 or 4 mM of TP; with decreasing concentrations of TP from 0.4 to 0.1 mM, more and more cell-cell junctions showed a FtsZ-CFP fluorescence. With 0.1 mM of TP, a condition allowing cells to grow, almost 52.9% of the cell-cell junctions have persistent FtsZ-CFP fluorescence, even higher than the mutant without TP, possibly because the mutant cell could no longer grow and lyse rapidly under such conditions (Table 1).

**TABLE 1:** FtsZ-ring formation and PG synthesis pattern in the wild type (WT) and strain TRS-*mraY*.

	Strains and conditions (TP: mM)	Midcell (%)	Cell-cell junction (%)
FtsZ-ring formation	TRS-*mraY* with TP 0	30	44
	TRS-*mraY* with TP 0.1	51.3	52.9
	TRS-*mraY* with TP 0.2	59.3	46.5
	TRS-*mraY* with TP 0.4	50.6	31.6
	TRS-*mraY* with TP 1	52.6	23.7
	TRS-*mraY* with TP 4	48.6	26.9
	WT	53.6	23.7
PG synthesis	TRS-*mraY* with TP 0	52	69.5
	TRS-*mraY* with TP 0.1	50.3	68.8
	TRS-*mraY* with TP 0.2	40	80.5
	TRS-*mraY* with TP 0.4	37.6	71.2
	TRS-*mraY* with TP 1	36.2	70
	TRS-*mraY* with TP 4	8.97	76.3
	WT	12.6	71.7

*The localization of FtsZ-CFP, or PG synthesis probed with HADA staining, was analyzed according to the data as shown in Figure 5 and Supplementary Figure 3. One hundred and fifty cells were counted in TRS-mraY with TP 0, TP 0.1, and TP 0.2 mM. And 500 cells were counted in TRS-mraY with TP 0.4, TP 1, TP 4 mM, and WT.*

Although the TRS-*mraY* mutant failed to grow and rapidly lysed under non-permissive conditions (Figures 3–5), our data on FtsZ-CFP localization and HADA labeling in the TRS-*mraY* mutant in the presence of different concentrations of TP as inducer for *mraY* expression supported the results described above. With decreasing concentrations of the TP inducer, more and more cells with midcell HADA incorporation could be observed, indicating more persistent activity of PG synthesis at the division sites as *mraY* is downregulated. These observations provide an adequate explanation on the increase of cell width under similar conditions, as shown in Figures 3, 4.

### MraY Interacts With Several Proteins Involved in Peptidoglycan Synthesis

**FIGURE 6:**
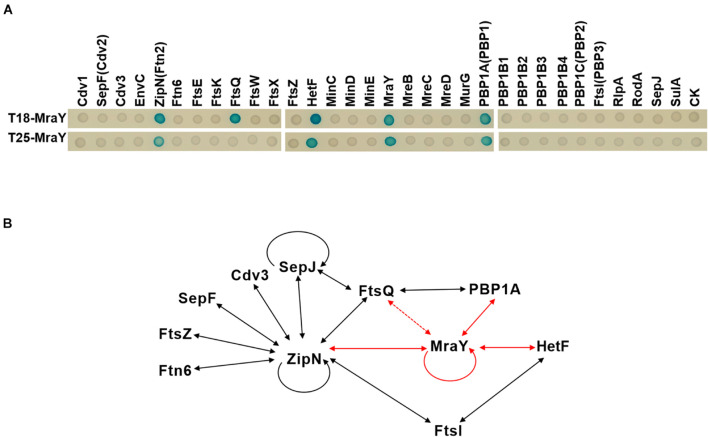
Protein-protein interactions involving MraY determined by bacterial two hybrid assays. **(A)** Two-hybrid analysis of MraY interaction partners. Interactions of given proteins fused to T25 and T18-MraY (top), or of given proteins fused to T18 and T25-MraY (bottom), were assayed in *E. coli* by observation of the β-galactosidase activity on agar plates. CK, empty vectors pKT25 or pUT18C. **(B)** Schematic representation of the web of protein-protein interactions among the 11 presently studied proteins involved in cell division or growth in *Anabaena*. Solid lines show protein-protein interactions detected with the BACTH system with both fusions of the same protein (in T18 and T25), dotted lines represent interactions detected with only one of the two fusions (either in T18, or T25 alone). The red arrows represent interactions detected in this study. Black arrows correspond to interactions reported elsewhere and further confirmed in the present study. See corresponding text in Results section for more detail.

In *E. coli*, elongasome and divisome share some common enzymes involved in PG synthesis such as PBP5, and components of the two complexes can even interact with each other (Mohammadi et al., 2007; Hugonnet et al., 2016). For example, MreB interacts with FtsZ in *E. coli*, and Lipid I and II synthesis enzymes including MraY are also found in both complexes (Hugonnet et al., 2016; Yoshii et al., 2019). Such interactions between the two complexes avoid competing PG synthesis activities and ensure cell growth and division in a spatiotemporal manner (Szwedziak and Löwe, 2013). Cyanobacterial divisome share some common components with those of other bacteria, but also has distinct features (Camargo et al., 2019; Xing et al., 2021). To understand the persistent PG synthesis activity following downregulation of MraY and its effect on the increase of cell width, we sought to determine whether MraY could interact directly with some components of the divisome or elongasome in *Anabaena*. For this purpose, MraY was expressed, respectively, in the two vectors of the bacterial two hybrid system (BACTH) and its interaction was tested with 31 proteins known to be involved in cell growth, division or elongation (Supplementary Table 1). MraY was found to interact with itself, as reported in other bacteria (White et al., 2010). As many cyanobacterial cell-division proteins (White et al., 2010; Camargo et al., 2019), MraY could interact with ZipN, a central scaffold of cyanobacterial divisome assembly. Another protein of the divisome, FtsQ, could also interact with MraY, at least when MraY was expressed from the T18 vector. In addition, we found that MraY also interacted with PBP1A (Figure 6), a component known as specific to the elongasome complex in *E. coli*. Interestingly, HetF, a newly identified component of the divisome in *Anabaena* necessary for heterocyst development, showed also interaction with MraY (Xing et al., 2021). HetF was required for septal PG synthesis under high-light conditions through interaction with FtsI. Thus, although we identified interaction between MraY and components of both elongasome and divisome (Figure 6), the interacting partners were different from those reported in other bacteria such as *E. coli* or *Caulobacter crescentus* (Mohammadi et al., 2007; White et al., 2010).

### MraY Is Involved in Heterocyst Development

**FIGURE 7:**
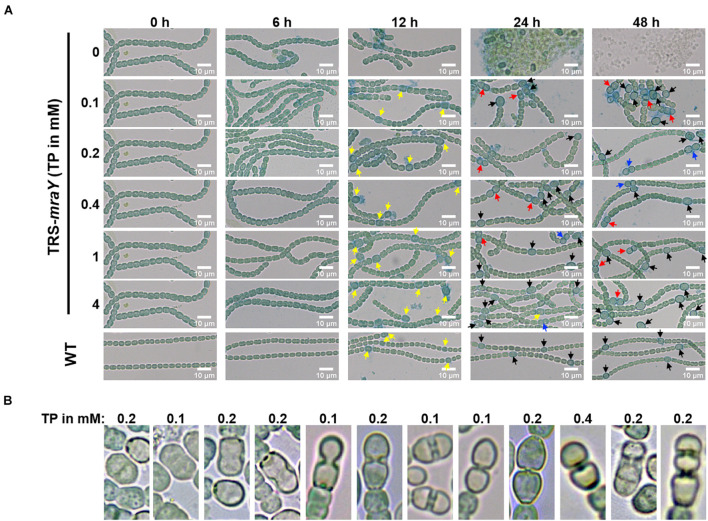
Heterocyst differentiation of TRS-*mraY* in BG11_0_ under permissive and non-permissive conditions. Samples of cell suspensions were directly observed under a light microscope and photographed at the indicated time. WT was used as the control. **(A)** Filaments of strain TRS-*mraY* grown in BG11 medium with 1 mM of TP were collected and transferred to BG11 medium without TP for 12 h (Supplementary Figure 6) to remove TP. Then filaments were collected and transferred to BG11_0_ medium with different concentrations of TP, in which they were incubated for indicated hours. Alcian blue stains exo-polysaccharides of proheterocysts or heterocysts. Yellow arrow, immature or proheterocysts; red arrow, aberrant heterocysts; blue arrow, double heterocysts; black arrow, mature heterocysts. **(B)** The morphology of aberrant heterocysts of strain TRS-*mraY* grown in BG11_0_ medium with different concentrations of TP.

**FIGURE 8:**
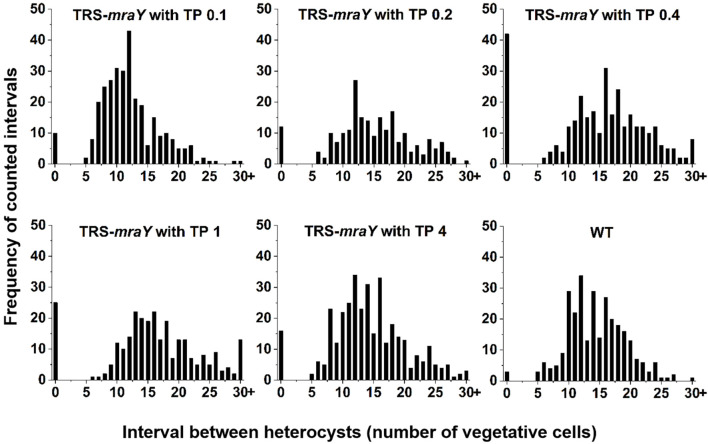
Heterocyst pattern in WT and TRS-*mraY* mutant. The same cultures as used in Figure 7A, at 48 h after transfer to BG11_0_, were analyzed. The frequency of intervals (number of vegetative cells between two heterocysts) is shown. When the interval is 0, it corresponds to double heterocysts as indicated in Figure 7. 350–450 heterocysts were counted for each condition.

Next, we examined the effect on heterocyst development with different concentrations of inducer in the conditional mutant strain TRS-*mraY*. In the WT used as a control, mature heterocysts with distinct morphology were formed about 24 h after the deprivation of combined nitrogen (Figure 7). At 12 h after the heterocyst induction, proheterocysts, enlarged cells that can be weakly stained with alcian blue, an agent specific for heterocyst-specific polysaccharide layer, could be revealed. Under non-permissive conditions in TRS-*mraY* mutant, because of the extensive fragmentation, we could only examine proheterocyst formation with alcian blue staining at 12 h. At that time point, no alcian-blue stained cells with cell enlargement could be found. At 24 h, most cells lysed, and no single heterocysts which are usually more resistant to cell lysis, could be identified. However, even with a low level of TP (0.1 mM), heterocyst development could be found despite more and more cell fragmentation occurred with time. At 48 h, free and resistant heterocysts detached from the filament due to fragmentation could be observed. Heterocyst formation was found with the addition of different concentrations of TP tested, and the distribution of heterocyst pattern was similar to WT (Figure 8). However, a high level of aberrant hetercoysts were observed in the mutant, especially at 0.1 mM of TP in which such aberrant heterocysts account up to 16.5% of all heterocysts formed after 48 h of nitrogen starvation (Table 2). With the addition of 1 or 4 mM of TP, the frequency of such aberrant heterocysts reduced to only about 3.5%. When closely examined, some aberrant heterocysts appeared to be at various stages of cell division (Figure 7B). Indeed, some of them had a large septum just formed, while others were more or less constricted, with a long-neck connecting the two cells. These doublets rather resemble those already observed in a conditional mutant of *polA* encoding DNA polymerase I (PolI) under non-permissive conditions where problem in DNA segregation prevented septum closure (Xing et al., 2020).

**TABLE 2:** Percentage of aberrant heterocysts of strain TRS-*mraY* grown in BG11_0_ medium.

Strains and conditions (TP: mM)	Percentage of aberrant heterocyst (%)		
	12 h	24 h	48 h
TRS-*mraY* with TP 0.1	1	9.12	16.5
TRS-*mraY* with TP 0.2	1.5	11.38	12.38
TRS-*mraY* with TP 0.4	1	7.75	8.25
TRS-*mraY* with TP 1	0.5	2.5	4
TRS-*mraY* with TP 4	0	2.25	3.5
WT	0	0	0

*Wild type (WT) was used as the control. 200 heterocysts were counted at 12 h, and 800 heterocysts were counted at 24 and 48 h.*

## Discussion

Bacteria have multiple redundant enzymes involved in PG synthesis. In *Anabaena*, some of the PBPs influence filament length and heterocyst envelope structures (Leganés et al., 2005; Burnat et al., 2014). Because of such redundancy, we studied the essential function of MraY, a protein required for the synthesis of lipid I, a precursor of PG synthesis, in order to examine the role of PG synthesis in the developmental cyanobacterium *Anabaena*. We therefore constructed a conditional mutant and demonstrated the essentiality of the *mraY* gene in *Anabaena*. Under permissive conditions, namely in the presence of 1 mM of TP, the cells grew nearly as well as WT, and the other phenotypes were also close to those found in the WT control. The absence of *mraY* induction resulted in some phenotypes as expected, such as rapid cell lysis and filament fragmentation, indicating the essential roles of PG in maintaining cell and filament integrity, as expected from the roles ascribed to PG in bacteria. Genetic analysis of the mutant also revealed interesting phenotypes in cell shape, coordination of PG synthesis and heterocyst development. When *mraY* expression becomes limiting, with low concentrations of inducer, cell length was little affected, but cell width increased as the concentration of the inducer decreasing (Figure 4). This phenotype can be explained by the persistent PG synthesis activity revealed by HADA labeling at the division sites. During the cell cycle of *Anabaena*, we have shown previously that in the first stage, FtsZ assembles first at the division site to initiate cell division (Zhang et al., 2018). In the second stage, HADA fluorescence signals and FtsZ-rings are localized together at the division sites, with some of them starting cell constriction. At the end, FtsZ could no longer be detected at the cell-cell junctions where HADA fluorescence could still be found. When similar experiments were carried out with strain TRS-*mraY* under non-permissive conditions or in the presence of low levels of TP, our results revealed a delay in the disassembly of FtsZ, as well as a persistent activity of HADA incorporation at the division sites. Consequently, septal PG synthesis becomes abnormally higher, leading to an increase of cell width.

Although bacterial PG synthesis is dependent on two protein complexes, the elongasome at the lateral cell wall, and divisome at the septal sites, multiple mechanisms have been found to coordinate PG synthesis in time and space. MraY and Mur proteins are found in both complexes. In *Anabaena*, it has been shown that when septal PG synthesis was inhibited by the antibiotic aztreonam, stronger lateral HADA incorporation, hence a stronger PG synthesis, along the side wall was found, leading to cell elongation (Zhang et al., 2018). Yfr1, a strictly conserved small RNA in cyanobacteria who has a negative regulation to *mraY*, is an important regulator of cell wall homeostasis and correct cell wall remodeling during heterocyst differentiation (Brenes-Álvarez et al., 2020). Overexpression of Yfr1 leads to wider and frequently nascent septa in the middle of cells that had not finished their previous division (Brenes-Álvarez et al., 2020). Here we show that when *mraY* expression becomes limiting, PG synthesis is more shifted toward the septal sites, resulting in a delay of cell division and a change of cell shape. These phenotypes are different from those observed with aztreonam treatment (Zhang et al., 2018). These results suggest that when lipid I is limiting, cells delay and ensure septal PG synthesis, while limiting lateral growth, so that cells can continue to proliferate.

The coordination of PG synthesis between the divisome and elongasome may be dependent on their interaction with MraY. While MraY interacts with components of both complexes in *Anabaena*, their interacting partners appear to be different. In other bacteria, for example, MraY interacts with MreB and RodA (Mohammadi et al., 2007; White et al., 2010) in the elongasome complex, and PBP2 and PBP3 (FtsI) in the divisome (Mohammadi et al., 2007; White et al., 2010; Szwedziak and Löwe, 2013). None of these interactions was confirmed for *Anabaena* by BACTH assays. However, we identified PBP1A of the elongasome complex, and FtsQ, ZipN and HetF in the divisome complex as its interacting partners. Except ZipN which acts as a central organizer of *Anabaena* divisome by interacting with most of the divisome components tested, other proteins are directly involved in PG synthesis in both complexes. FtsW and FtsI form a PG synthase in the divisome whose activity is positively regulated by FtsQLB subcomplex (Marmont and Bernhardt, 2020). HetF is a specific component of heterocyst-forming cyanobacteria and interacts directly with FtsI (Xing et al., 2021). Thus, although the presence of MraY in both complexes is conserved for coordination of PG synthesis, the precise mechanism differs in *Anabaena*.

*mraY* has a complex promoter region in which one transcription start site is specifically upregulated in heterocysts (Muro-Pastor et al., 2017), suggesting a role of this enzyme in heterocyst differentiation. Under non-permissive conditions, the strain TRS-*mraY* gives no sign of detectable heterocyst development, by staining with alcian blue. Since the mutant exhibited rapid filament fragmentation and cells lysis, it was impossible to follow the entire course of heterocyst differentiation. However, the interaction between MraY and HetF which is required for heterocyst development and cell division, suggests a possible link between PG synthesis and heterocyst differentiation. This is in agreement with previous finding indicating that some members of the PBPs is necessary for PG layer formation or maintenance in heterocysts, thus for functional heterocyst formation (Lázaro et al., 2001; Leganés et al., 2005). It is also consistent with the enhanced HADA incorporation observed during heterocyst formation (Zhang et al., 2018; Wang et al., 2021). We found that even a low level of inducer added to strain TRS-*mraY* could restore heterocyst formation, but led to a high percentage of doublets heterocysts. Unlike contiguous heterocysts observed in *patS* mutant, or *hetR* overexpression strains, these heterocyst doublets have an abnormal neck connecting them. They appear to be at the different stages of cell constriction, which resemble those found in the *polA* mutant caused by incomplete DNA segregation and consequently failure of septum closure, or those reported for a conditional *patS patX hetN* tripple mutant (Khudyakov et al., 2020). These heterocyst doublets are likely the results of delayed cell division, with persistent PG synthesis at the division site. In such a case, a cell started with differentiation continues to divide slowly, and responds to developmental signals together because of the shared cell space, resulting in the formation of differentiated doublets in the end. Therefore, such a phenotype is a consequence of a misregulation of cell growth and division when *mraY* expression is limiting.

We and other laboratories have examined so far different events of the cell cycle in relationship to heterocyst development, including cell growth as in the case of the TRS-*mraY* conditional mutant reported here, DNA replication (Xing et al., 2020), and cell division (Sakr et al., 2006; Springstein et al., 2020; Valladares et al., 2020; Xing et al., 2021). It appears that some steps of cell division are the key connection between cell cycle and heterocyst differentiation. HetF and SepI are direct components of the divisome (Springstein et al., 2020; Xing et al., 2021), and PatA also influences cell division (Valladares et al., 2020). FtsZ is a dual functional protein for both cell division and the regulation of heterocyst development (Wang et al., 2021). The enhanced activity of PG synthesis during heterocyst constitutes one interesting point for our further understanding of cell division and heterocyst development in *Anabaena*.

## Materials and Methods

### Strains and Growth Conditions

All strains used in this study are listed in Supplementary Table 2. *Anabaena* strains were cultivated in BG11 (Stanier et al., 1971) or BG11_0_ (BG11 without nitrate) in a shaker (30°C at 180 rpm) with illumination at 30 μmol photons m^–2^ s^–1^. In order to maintain TRS-*mraY*, the TRS-*mraY* mutant was cultivated and stored in BG11 or BG11_0_ with 1 mM of theophylline (TP). 100 μg ml^–1^ neomycin, or of 5 μg ml^–1^ spectinomycin and 2.5 μg ml^–1^ streptomycin, were added to the cultures when needed. Absorbance at 750 nm was measured at the indicated times upon inoculation in BG11 or BG11_0_ medium to measure the growth curve of different strains.

### RNA Isolation and RT-PCR

RNA was isolated from filaments of *Anabaena* strains grown in BG11 medium for 5 days. RNA (20 ng) was used for reverse transcription with the Quantitec Reverse Transcription kit (Qiagen). To check if *asl4317*, *all4316* (*mraY*), and *all4315* could be cotranscribed, the obtained cDNA was used for RT-PCR, using oligonucleotide primers covering the intergenic regions. The primer pairs used were: Pall4316F280m/Pall4316R220, Pall4315F452m/Pall4315R48, Pall4315F1702m/Pall4315R48, respectively (Supplementary Table 3). The number of cycles at which the PCR reaction was in the exponential range was empirically determined. Samples were taken and analyzed by electrophoresis in agarose gels.

### Plasmid and Strain Constructions

All oligonucleotide primers are listed in Supplementary Table 3. All employed plasmids (Supplementary Table 4) generated in this study were verified by Sanger sequencing. To construct TRS-*mraY* strain, plasmid pTRS-*mraY*R10m-sp was first generated in a similar way as previously described using pCpf1-sp plasmid (Niu et al., 2018; Xing et al., 2021). The repair template, in which the weak and native promoter of *mraY* was replaced by a tunable synthetic riboswitch inducible by TP, was generated by fusing the upstream and downstream region of the target sequence using primers listed in Supplementary Table 3 (Pall4316F970m/Pall4316R40m, Priboswitch2/Pall4316F1c/Pall4316R1140). The spacer sequence was designed according to the described rules (Niu et al., 2018) and prepared by annealing two complementary primers (cr_all4316R10mF/cr_all4316R10mR). To generate the plasmid that knocks out a specific sequence, the respective repair template and the spacer sequence were sequentially cloned into pCpf1-sp at the sites of *Bgl*II-*Bam*HI and *Aar*I-*Aar*I. For plasmid pP*_*mraY*_-mraY*, upstream (native promoter of *asl4317* and *mraY*) and downstream (*mraY*) sequences were amplified from *Anabaena* gDNA using primers Pasl4317F243m/Pasl4317R1m and Pall4316F1f/Pall4316R1107b, respectively, and ligated into linearized pCT (using primers PpCT-R2979/PV_20) by Gibson assembly. For plasmid pP*_*coaT*_-mraY*, upstream (Co^2+^ or Zn^2+^ inducible promoter from *Synechocystis* sp. PCC 6803) sequence was amplified from *Synechocystis* sp. PCC 6803 gDNA using primers Pslr0797F1193ma/Pslr0797R1mb, and downstream (*mraY*) sequence was amplified from *Anabaena* gDNA using primers Pall4316F1g/Pall4316R1107b, respectively, and these two fragments were ligated into linearized pCT (using primers PpCT-R2979/PV_20) by Gibson assembly.

Plasmid pTRS-*mraY*R10m-sp was transferred by conjugation to WT or the *ftsZ-cfp* strain with selection for Sm^*r*^ Sp^*r*^ to get TRS-*mraY* and *ftsZ-cfp*:TRS-*mraY* strain, respectively. Plasmids pP*_*mraY*_-mraY* and pP*_*coaT*_-mraY* were transferred by conjugation to TRS-*mraY* mutant strain with selection for Nm^*r*^ to get TRS-*mraY*:P*_*mraY*_-mraY* and TRS-*mraY*:P*_*coaT*_-mraY* strain, respectively.

### Bacterial Adenylate Cyclase Two-Hybrid System Kit Assay

The Bacterial Adenylate Cyclase Two-Hybrid System Kit (BATCH) based on the reconstitution of adenylate cyclase was used for testing protein-protein interaction (Battesti and Bouveret, 2012). 32 genes in this study were amplified with primers listed in Supplementary Table 3, and individual PCR products were assembled into linearized pUT18C and pKT25 vectors. All the resulting plasmids were verified by PCR and Sanger sequencing. The plasmids were co-transformed into strain BTH101, and the transformants were plated on solid LB medium containing 50 μg L^–1^ ampicillin, 25 μg L^–1^ kanamycin, 0.5 mM L^–1^ IPTG, and 40 μg L^–1^ 5-bromo-4-chloro-3-indolyl-b-D-galactopyranoside (X-gal) to estimate the strength of interactions.

### Microscopy

A Sdptop EX30 microscope was used to take bright-field images, and an Sdptop EX40 epifluorescence microscope was used to take fluorescence images. A filter [exciter (EX) 379–401, dichroic beamsplitter (DM) 420LP, emitter (EM) 435–485] was used to image HADA fluorescence (exposure time of 200 ms). A filter (EX426-446, DM455LP, EM460-500) was used to image CFP fluorescence (exposure time of 1 s). Fluorescence images were taken with an oil immersion lens objective (100/1.28). All images were processed using ImageJ without deconvolution. Cell length, cell width, cell area and filament length (cells per filament) were analyzed using the ImageJ software of microscopy images. Statistical tests and plotting of data were performed with the GraphPad Prim software or Origin.

### HADA Labeling

For HADA labeling, *Anabaena* WT was grown in BG11 liquid medium, and TRS-*mraY* mutant was grown in BG11 liquid medium with 1 mM of TP. WT and TRS-*mraY* were washed three times in BG11 and incubated with 150 μM of HADA in BG11 without TP or with different concentrations of TP to observe PG synthesis for 24 h at standard growth conditions by microscopy.

## Data Availability Statement

The original contributions presented in the study are included in the article/Supplementary Material, further inquiries can be directed to the corresponding author/s.

## Author Contributions

JL and W-YX performed the experiments. J-YZ, XZ, YY, and C-CZ designed the experiments. C-CZ and JL wrote the manuscript. All authors contributed to the article and approved the submitted version.

## Conflict of Interest

The authors declare that the research was conducted in the absence of any commercial or financial relationships that could be construed as a potential conflict of interest.

## Publisher’s Note

All claims expressed in this article are solely those of the authors and do not necessarily represent those of their affiliated organizations, or those of the publisher, the editors and the reviewers. Any product that may be evaluated in this article, or claim that may be made by its manufacturer, is not guaranteed or endorsed by the publisher.
