# Supplementary material for: Functions of the Essential Gene mraY in Cellular Morphogenesis and Development of the Filamentous Cyanobacterium Anabaena PCC 7120
Source: Front Microbiol. 2021 Oct 21;12:765878. doi: 10.3389/fmicb.2021.765878 (PMC8566892; doi:10.3389/fmicb.2021.765878)
Supplement: Supplementary file 1 [file Data_Sheet_1.docx]

SUPPLEMENTAL MATERIALS

TABLE S1 List of proteins expressed for bacterial two-hybrid assay. *, the signal peptide of the corresponding protein is removed.

| NO. | Protein name | Gene number (Anabaena PCC 7120) |
| --- | --- | --- |
| 1 | Cdv1 | *all4287* |
| 2 | SepF (Cdv2) | *alr0487* |
| 3 | Cdv3 | *alr4701* |
| 4 | EnvC | *all7672* |
| 5 | ZipN (Ftn2) | *all2707* |
| 6 | Ftn6 | *all1616* |
| 7 | FtsE | *alr1706* |
| 8 | FtsK | *all7666* |
| 9 | FtsQ | *alr3857* |
| 10 | FtsW | *all0154* |
| 11 | FtsX | *all1757* |
| 12 | FtsZ | *alr3858* |
| 13 | HetF | *alr3546* |
| 14 | MinC | *alr3455* |
| 15 | MinD | *alr3456* |
| 16 | MinE | *asr3457* |
| 17 | MraY | *all4316* |
| 18 | MreB | *all0087* |
| 19 | MreC* | *all0086* |
| 20 | MreD | *all0085* |
| 21 | MurG | *alr0477* |
| 22 | PBP1A (PBP1) | *all2952* |
| 23 | PBP1B1 | *all2981* |
| 24 | PBP1B2 | *alr4579* |
| 25 | PBP1B3 | *alr5324* |
| 26 | PBP1B4 | *alr5326* |
| 27 | PBP1C (PBP2)* | *alr5101* |
| 28 | FtsI (PBP3) | *alr0718* |
| 29 | RlpA* | *alr2935* |
| 30 | RodA | *alr0653* |
| 31 | SepJ | *alr2338* |
| 32 | SulA | *all2390* |

TABLE S2 Strains used in this study.

| Strains | Description | Source |
| --- | --- | --- |
| *Anabaena* PCC 7120 | Wild type | Pasteur Culture Collection |
| TRS-*mraY* | *mraY* conditional mutant | This study |
| TRS-*mraY*::P*_mraY_*-*mraY* | TRS-*mraY* bearing pP*_mraY_*-*mraY*; Nm^r^ | This study |
| TRS-*mraY*::P*_coaT_*-*mraY* | TRS-*mraY* bearing pP*_coaT_*-*mraY*; Nm^r^ | This study |
| *ftsZ-cfp* | *ftsZ-cfp* translational fusion on chromosome; Nm^r^ | 4 |
| *ftsZ*-*cfp*::TRS-*mraY* | *mraY* conditional mutant with *ftsZ-cfp*  translational fusion on chromosome; Nm^r^ | This study |

**TABLE S3** Primers used in this study.

| Primers | Sequence (5’-3’) |
| --- | --- |
| Pall4316F970m | GCAGAAATTCGATATCTAGATCTGTACCCGATTAATCGTTGTAAACCCA |
| Pall4316R40m | AAGGGCATCAAGACGATGCTGGTATCACCTATAGGGACTATATATAACAGAGTCCCAGT |
| Pall4316F1c | TGCTAAGGAGGTAACAACAAGGTGGACGCTAAATTATCGCCTAATCAAG |
| Pall4316R1140 | CGCAACGTTGTTGCCATTGCTTTGTGAAATATTCAGAATGGGGCGATGA |
| Priboswitch2 | TCTTGTTGTTACCTCCTTAGCAGGGTGCTGCCAAGGGCATCAAGACGATGCT |
| cr_all4316R10mF | AGATCCTTCACTCCACACTTTAATAA |
| cr_all4316R10mR | AGACTTATTAAAGTGTGGAGTGAAGG |
| Pall4316F1047m | ATCACTTTTAATCAGTACATCTAAAGCAGT |
| Pall4316R1m | AGCGCTACCGACGCTAGTGCTAAAAGTTTCCCTTCACTCCACACTT |
| PV-19 | CATCTTGTTGTTACCTCCTTAGCA |
| Pall4316F280m | CCAGCGTGCGGCTGTCATCCCT |
| Pall4316R220 | TTTTCAGTGCTTGCAGTAGAGGCA |
| Pall4315F452m | GCCATCTTAGCCGCCATCTG |
| Pall4315R48 | AATAGTAGTAATTCCAGCTAAGAATATAGACGA |
| Pall4315F1702m | AACCAGATTTTTCCGATGAAGAAGA |
| PpCT-R2979 | TCCAAAAAAAAACCCCGCCGAA |
| PV_20 | GGGGGTTCTGGTGGTGGTAGCACT |
| Pasl4317F243m | CGGCGGGGTTTTTTTTTGGACACATTCCTTTAACAAGCGTCAT |
| Pasl4317R1m | ATCGATATCTAATGAGCAAAAAGACTTATG |
| Pall4316F1f | TTTGCTCATTAGATATCGATGTGGACGCTAAATTATCGCCTA |
| Pall4316R1107b | ACCACCAGAACCCCCTGCTCCACCGGAGGCGATCGCCAGACAGAT |
| Pslr0797F1193ma | CGGCGGGGTTTTTTTTTGGATAAAGACAAGTGAGATAGCAGTGG |
| Pslr0797R1mb | GCTTTTTAACTTGGATTTTTACCTTCT |
| Pall4316F1g | AAAAATCCAAGTTAAAAAGCGTGGACGCTAAATTATCGCCTA |
| PpKT25a-F733 | GAGATCTAGATCGACATCTGTCGAGC |
| PpKT25a-R720 | TTCACCACTAGAGGTGATCA |
| Pall4287F1 | ATCACCTCTAGTGGTGAAGTGCAAGGCGGCGACCCT |
| Pall4287R414 | GATGTCGATCTAGATCTCCTATTGAGGAACTTTCAAGTTCTC |
| Palr4701F1 | ATCACCTCTAGTGGTGAAGTGTTTCAGGATGCAGCTGCAAC |
| Palr4701R467 | GATGTCGATCTAGATCTCTACAGTAACTCAAGTATCAGCGT |
| Pall7672F1 | ATCACCTCTAGTGGTGAAATGCGAATAATCCTTGAAACTGACG |
| Pall7672R651 | GATGTCGATCTAGATCTCTCAACGGTTTGCTAAATATTCGTGT |
| Pall2707F1b | ATCACCTCTAGTGGTGAAATGTTGATCACGGTGCAGGGGA |
| Pall2707R2397 | GATGTCGATCTAGATCTCTTAATTTATAGCGGCTGACATCC |
| Pall1616F1b | ATCACCTCTAGTGGTGAAATGCGATGGGTCGATGATTC |
| Pall1616R684b | GATGTCGATCTAGATCTCTTAGGAATTTTCGTTAGATATTGCT |
| Palr1706F1 | ATCACCTCTAGTGGTGAAATGGTGCAGTTAAATGGGGTGAC |
| Palr1706R680 | GATGTCGATCTAGATCTCGGTTTCTCATTTGTAATCCCTGAC |
| Pall7666F1 | ATCACCTCTAGTGGTGAAATGTTAGAAATGTATATTAATAACAAC |
| Pall7666R2196 | GATGTCGATCTAGATCTCGGTAAAGGCATGGTTAGTTCAA |
| Palr3857F1 | ATCACCTCTAGTGGTGAAATGCCTGGTATAGCGTCGGT |
| Palr3857R846 | GATGTCGATCTAGATCTCTTATGGCGTTTGATTTTTAATTGTCTC |
| Pall1757F1 | ATCACCTCTAGTGGTGAAGTGTTTAAATTTCTCACGAAACTTG |
| Pall1757R903 | GATGTCGATCTAGATCTCCTAACTTTTGGCAAAACGTCG |
| Palr3455F1 | ATCACCTCTAGTGGTGAAATGGTGGATGAGCAGGTCA |
| Palr3455R894 | GATGTCGATCTAGATCTCTTATGGTGTCTGATTGATCTTACT |
| Palr3456F1 | ATCACCTCTAGTGGTGAAATGACTCGCATTATTGTAATTACCT |
| Palr3456R807 | GATGTCGATCTAGATCTCTTACACAATCTTAGTCCATAACAACC |
| Pasr3457F1 | ATCACCTCTAGTGGTGAAATGATCCTCGAATTATTAGACAAACTC |
| Pasr3457R341 | GATGTCGATCTAGATCTCGTGTTGTCAATTGACAAACTCG |
| Pall4316F1 | ATCACCTCTAGTGGTGAAGTGGACGCTAAATTATCGCCTA |
| Pall4316R1149 | GATGTCGATCTAGATCTCATGATTAATTTTGTGAAATATTCAGAATGG |
| Pall0087F1 | ATCACCTCTAGTGGTGAAGTGGGGCTTTTTAGGAACTTTCG |
| Pall0087R1044 | GATGTCGATCTAGATCTCCTACATATTTCGAGATCGTCCGCTA |
| Pall0086F88 | ATCACCTCTAGTGGTGAACAGACTCAAGGCGGGTTTTTAGCGGAA |
| Pall0086R822 | GATGTCGATCTAGATCTCCTAGTTGGACTTTTGTTGCTGTGA |
| Pall0085F1 | ATCACCTCTAGTGGTGAATTGCTTCACCTACTCGATTG |
| Pall0085R513 | GATGTCGATCTAGATCTCCTAACTTTCCAACATCTTCATTCT |
| Palr0477F1 | ATCACCTCTAGTGGTGAAGTGACTACAGGTGGATATATCGC |
| Palr0477R786 | GATGTCGATCTAGATCTCTCATCTCTCCACAACTTCCCGTA |
| Pall2952F1 | ATCACCTCTAGTGGTGAAGTGGTAAGACAAAAAAAATTATTAAACC |
| Pall2952R2294 | GATGTCGATCTAGATCTCCCAGCTATAATAGCTCACAACCTA |
| Pall2981F1 | ATCACCTCTAGTGGTGAAGTGTCGTCTAGGACTTTTGAACA |
| Pall2981R1969 | GATGTCGATCTAGATCTCAGGGAAGAGAAAGTGACCAAT |
| Palr4579F1 | ATCACCTCTAGTGGTGAAATGAATTCCCCCCAACCCCCTCAC |
| Palr4579R2284 | GATGTCGATCTAGATCTCGTGGGGAGAGTTTTTATCTCGTA |
| Palr5324F1 | ATCACCTCTAGTGGTGAAGTGTCTTCATTAAGGACTTTTGA |
| Palr5324R1982 | GATGTCGATCTAGATCTCGGTTGAAATTAAATTTGGTGCTAC |
| Palr5326F1 | ATCACCTCTAGTGGTGAAGTGTCTTCATCAGAAAATATTGAACAG |
| Palr5326R1961 | GATGTCGATCTAGATCTCTTCAATTAGCCATAACATCATCCAA |
| Palr2935F76 | ATCACCTCTAGTGGTGAACAAACTACTAAAGCTACAGCACCA |
| Palr2935R1116 | GATGTCGATCTAGATCTCCTATCTTCCCAAAACTTCAATCCTG |
| Palr0653F1 | ATCACCTCTAGTGGTGAAATGTTATTAAAACGATCGCTTCCCA |
| Palr0653R1317 | GATGTCGATCTAGATCTCTCAAAAATAATATTTCTGTCGTTGTCG |
| Palr2338F1 | ATCACCTCTAGTGGTGAAATGGGGCGATTTGAGAAG |
| Palr2338R2276 | GATGTCGATCTAGATCTCAAGTCCTTTGTCATAAGGGTT |
| Pall2390F1 | ATCACCTCTAGTGGTGAAATGAAAGTAGCAATTACAGGCG |
| Pall2390R921 | GATGTCGATCTAGATCTCTCACTGCAAAATTTCTTGTAAAGC |

TABLE S4 Plasmids used in this study.

| Plasmids | Description | Source |
| --- | --- | --- |
| pCT | Km^r^Nm^r^; | 1, 2 |
| pCpf1-sp | Sm^r^ Sp^r^; CRISPR-Cpf1-Based Genome Editing vector | 1 |
| pTRS-*mraY*R10m-sp | Sm^r^ Sp^r^; CRISPR-Cpf1 editing plasmid for *mraY* conditional mutant | This study |
| pP*_mraY_*-*mraY* | Km^r^ Nm^r^; plasmid carrying *mraY* ORF and *mraY* promoter, used for complement | This study |
| pP*_coaT_*-*mraY* | Km^r^ Nm^r^; plasmid carrying *mraY* ORF and *coaT* promoter, used for complement | This study |
| pKT25 | Km^r^; empty vector (encoding the T25 fragment), used for BACTH assay | 3 |
| pUT18C | Car^r^; empty vector (encoding the T18 fragment), used for BACTH assay | 3 |
| pKT25-*zipA* | Km^r^; positive control, used for BACTH assay | 3 |
| pUT18C-*zipA* | Car^r^; positive control, used for BACTH assay | 3 |
| pKT25-*cdv1* | Km^r^; pKT25 carrying *cdv1* ORF, used for BACTH assay | This study |
| pUT18C-*cdv1* | Car^r^; pUT18C carrying *cdv1* ORF, used for BACTH assay | This study |
| pKT25-*sepF* | Km^r^; pKT25 carrying *sepF* ORF, used for BACTH assay | 4 |
| pUT18C-*sepF* | Car^r^; pUT18C carrying *sepF* ORF, used for BACTH assay | 4 |
| pKT25-*cdv3* | Km^r^; pKT25 carrying *cdv3* ORF, used for BACTH assay | This study |
| pUT18C-*cdv3* | Car^r^; pUT18C carrying *cdv3* ORF, used for BACTH assay | This study |
| pKT25-*envC* | Km^r^; pKT25 carrying *envC* ORF, used for BACTH assay | This study |
| pUT18C-*envC* | Car^r^; pUT18C carrying *envC* ORF, used for BACTH assay | This study |
| pKT25-*zipN* | Km^r^; pKT25 carrying *zipN* ORF, used for BACTH assay | This study |
| pUT18C-*zipN* | Car^r^; pUT18C carrying *zipN* ORF, used for BACTH assay | This study |
| pKT25-*ftn6* | Km^r^; pKT25 carrying *ftn6* ORF, used for BACTH assay | This study |
| pUT18C-*ftn6* | Car^r^; pUT18C carrying *ftn6* ORF, used for BACTH assay | This study |
| pKT25-*ftsE* | Km^r^; pKT25 carrying *ftsE* ORF, used for BACTH assay | This study |
| pUT18C-*ftsE* | Car^r^; pUT18C carrying *ftsE* ORF, used for BACTH assay | This study |
| pKT25-*ftsK* | Km^r^; pKT25 carrying *ftsK* ORF, used for BACTH assay | This study |
| pUT18C-*ftsK* | Car^r^; pUT18C carrying *ftsK* ORF, used for BACTH assay | This study |
| pKT25-*ftsQ* | Km^r^; pKT25 carrying *ftsQ* ORF, used for BACTH assay | This study |
| pUT18C-*ftsQ* | Car^r^; pUT18C carrying *ftsQ* ORF, used for BACTH assay | This study |
| pKT25-*ftsW* | Km^r^; pKT25 carrying *ftsW* ORF, used for BACTH assay | 4 |
| pUT18C-*ftsW* | Car^r^; pUT18C carrying *ftsW* ORF, used for BACTH assay | 4 |
| pKT25-*ftsX* | Km^r^; pKT25 carrying *ftsX* ORF, used for BACTH assay | This study |
| pUT18C-*ftsX* | Car^r^; pUT18C carrying *ftsX* ORF, used for BACTH assay | This study |
| pKT25-*ftsZ* | Km^r^; pKT25 carrying *ftsZ* ORF, used for BACTH assay | 4 |
| pUT18C-*ftsZ* | Car^r^; pUT18C carrying *ftsZ* ORF, used for BACTH assay | 4 |
| pKT25-*hetF* | Km^r^; pKT25 carrying *hetF* ORF, used for BACTH assay | 4 |
| pUT18C-*hetF* | Car^r^; pUT18C carrying *hetF* ORF, used for BACTH assay | 4 |
| pKT25-*minC* | Km^r^; pKT25 carrying *minC* ORF, used for BACTH assay | This study |
| pUT18C-*minC* | Car^r^; pUT18C carrying *minC* ORF, used for BACTH assay | This study |
| pKT25-*minD* | Km^r^; pKT25 carrying *minD* ORF, used for BACTH assay | This study |
| pUT18C-*minD* | Car^r^; pUT18C carrying *minD* ORF, used for BACTH assay | This study |
| pKT25-*minE* | Km^r^; pKT25 carrying *minE* ORF, used for BACTH assay | This study |
| pUT18C-*minE* | Car^r^; pUT18C carrying *minE* ORF, used for BACTH assay | This study |
| pKT25-*mraY* | Km^r^; pKT25 carrying *mraY* ORF, used for BACTH assay | This study |
| pUT18C-*mraY* | Car^r^; pUT18C carrying *mraY* ORF, used for BACTH assay | This study |
| pKT25-*mreB* | Km^r^; pKT25 carrying *mreB* ORF, used for BACTH assay | This study |
| pUT18C-*mreB* | Car^r^; pUT18C carrying *mreB* ORF, used for BACTH assay | This study |
| pKT25-*mreC* | Km^r^; pKT25 carrying mreC ORF that has been removed the signal peptide,  used for BACTH assay | This study |
| pUT18C-*mreC* | Car^r^; pUT18C carrying *mreC* ORFthat has been removed the signal peptide, used for BACTH assay | This study |
| pKT25-*mreD* | Km^r^; pKT25 carrying *mreD* ORF, used for BACTH assay | This study |
| pUT18C-*mreD* | Car^r^; pUT18C carrying *mreD* ORF, used for BACTH assay | This study |
| pKT25-*murG* | Km^r^; pKT25 carrying *murG* ORF, used for BACTH assay | This study |
| pUT18C-*murG* | Car^r^; pUT18C carrying *murG* ORF, used for BACTH assay | This study |
| pKT25-PBP1A | Km^r^; pKT25 carrying PBP1A ORF, used for BACTH assay | This study |
| pUT18C-PBP1A | Car^r^; pUT18C carrying PBP1A ORF, used for BACTH assay | This study |
| pKT25-PBP1B1 | Km^r^; pKT25 carrying PBP1B1 ORF, used for BACTH assay | This study |
| pUT18C-PBP1B1 | Car^r^; pUT18C carrying PBP1B1 ORF, used for BACTH assay | This study |
| pKT25-PBP1B2 | Km^r^; pKT25 carrying PBP1B2 ORF, used for BACTH assay | This study |
| pUT18C-PBP1B2 | Car^r^; pUT18C carrying PBP1B2 ORF, used for BACTH assay | This study |
| pKT25-PBP1B3 | Km^r^; pKT25 carrying PBP1B3 ORF, used for BACTH assay | This study |
| pUT18C-PBP1B3 | Car^r^; pUT18C carrying PBP1B3 ORF, used for BACTH assay | This study |
| pKT25-PBP1B4 | Km^r^; pKT25 carrying PBP1B4 ORF, used for BACTH assay | This study |
| pUT18C-PBP1B4 | Car^r^; pUT18C carrying PBP1B4 ORF, used for BACTH assay | This study |
| pKT25-*pbp2* | Km^r^; pKT25 carrying *pbp2* ORF that has been removed the signal peptide, used for BACTH assay | 4 |
| pUT18C-*pbp2* | Car^r^; pUT18C carrying *pbp2* ORF that has been removed the signal peptide, used for BACTH assay | 4 |
| pKT25-*ftsI* | Km^r^; pKT25 carrying *ftsI* ORF, used for BACTH assay | 4 |
| pUT18C-*ftsI* | Car^r^; pUT18C carrying *ftsI* ORF, used for BACTH assay | 4 |
| pKT25-*rlpA* | Km^r^; pKT25 carrying *rlpA* ORF that has been removed the signal peptide, used for BACTH assay | This study |
| pUT18C-*rlpA* | Car^r^; pUT18C carrying *rlpA* ORF that has been removed the signal peptide, used for BACTH assay | This study |
| pKT25-*rodA* | Km^r^; pKT25 carrying *rodA* ORF, used for BACTH assay | This study |
| pUT18C-*rodA* | Car^r^; pUT18C carrying *rodA* ORF, used for BACTH assay | This study |
| pKT25-*sepJ* | Km^r^; pKT25 carrying *sepJ* ORF, used for BACTH assay | This study |
| pUT18C-*sepJ* | Car^r^; pUT18C carrying *sepJ* ORF, used for BACTH assay | This study |
| pKT25-*sulA* | Km^r^; pKT25 carrying *sulA* ORF, used for BACTH assay | This study |
| pUT18C-*sulA* | Car^r^; pUT18C carrying *sulA* ORF, used for BACTH assay | This study |

*^a^* Km, kanamycin; Nm, neomycin; Sm, streptomycin; Sp, spectinomycin; Car: carbenicillin.


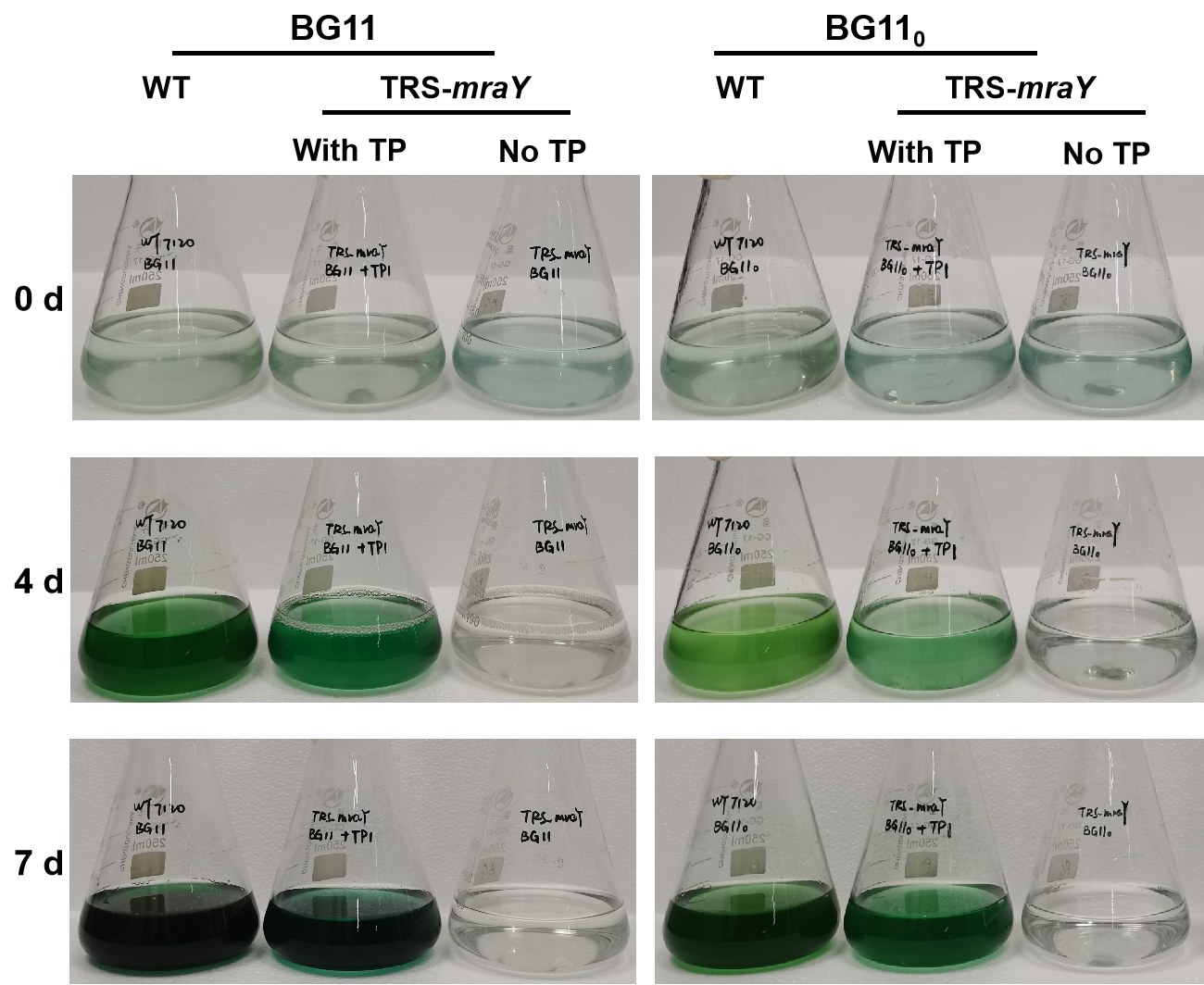


**Fig. S1:** The growth of TRS-*mraY* in the BG11 and BG11_0_ liquid medium under permissive and non-permissive conditions. *Anabaena* WT and TRS-*mraY* in the BG11 and BG11_0_ supplemented with 1 mM of theophylline (With TP) or without TP (No TP). Cultures were photographed after the indicated time of incubation.


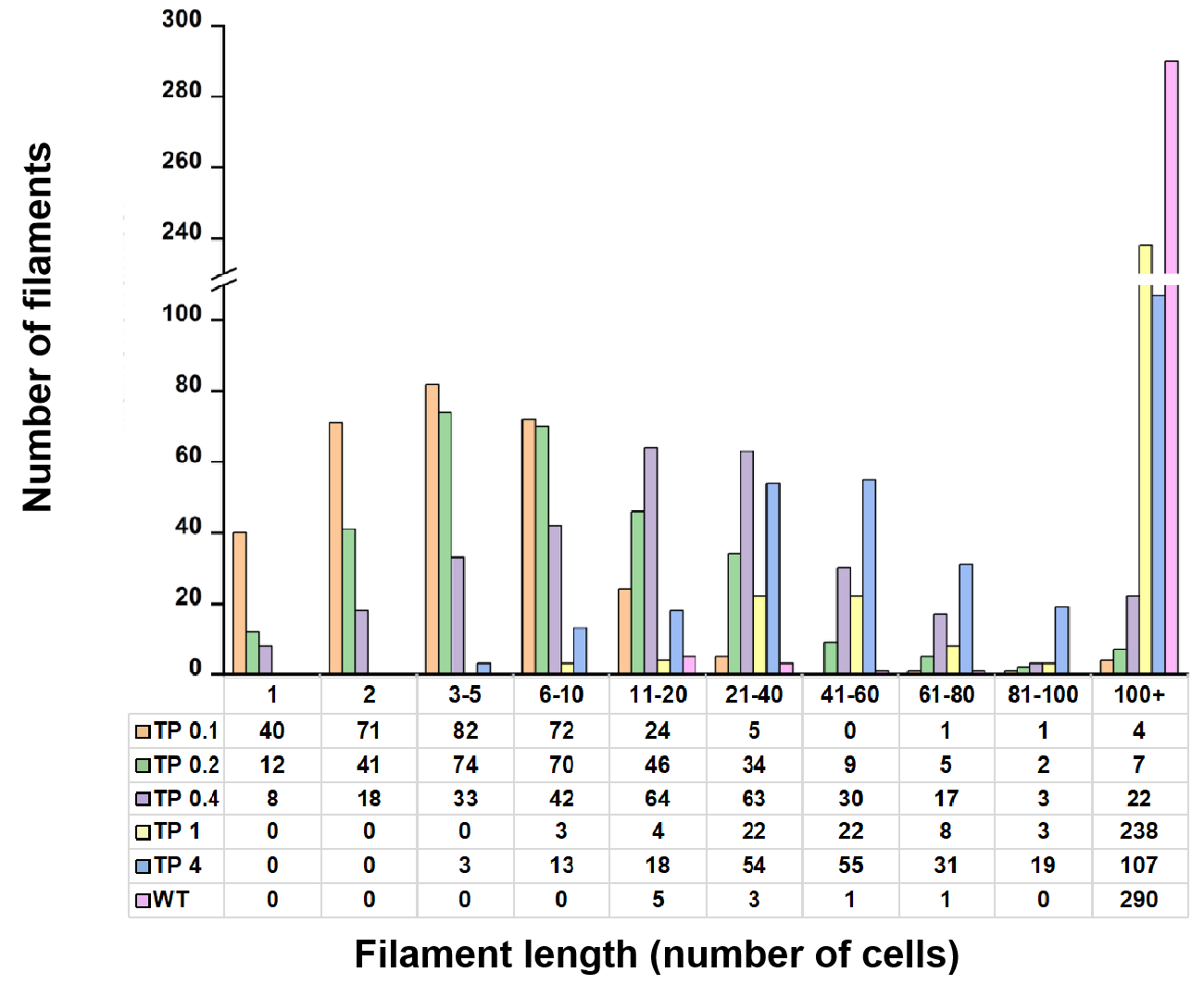


**Fig. S2:** MraY is involved in filament integrity. Filament length of WT and TRS-*mraY* mutant with different concentrations of TP were analysed after 4 days of culture. The filament length corresponds to the number of cells per filament. Filaments with more than 100 cells were counted as 100 cells per filament. 300 filaments were analysed.

**
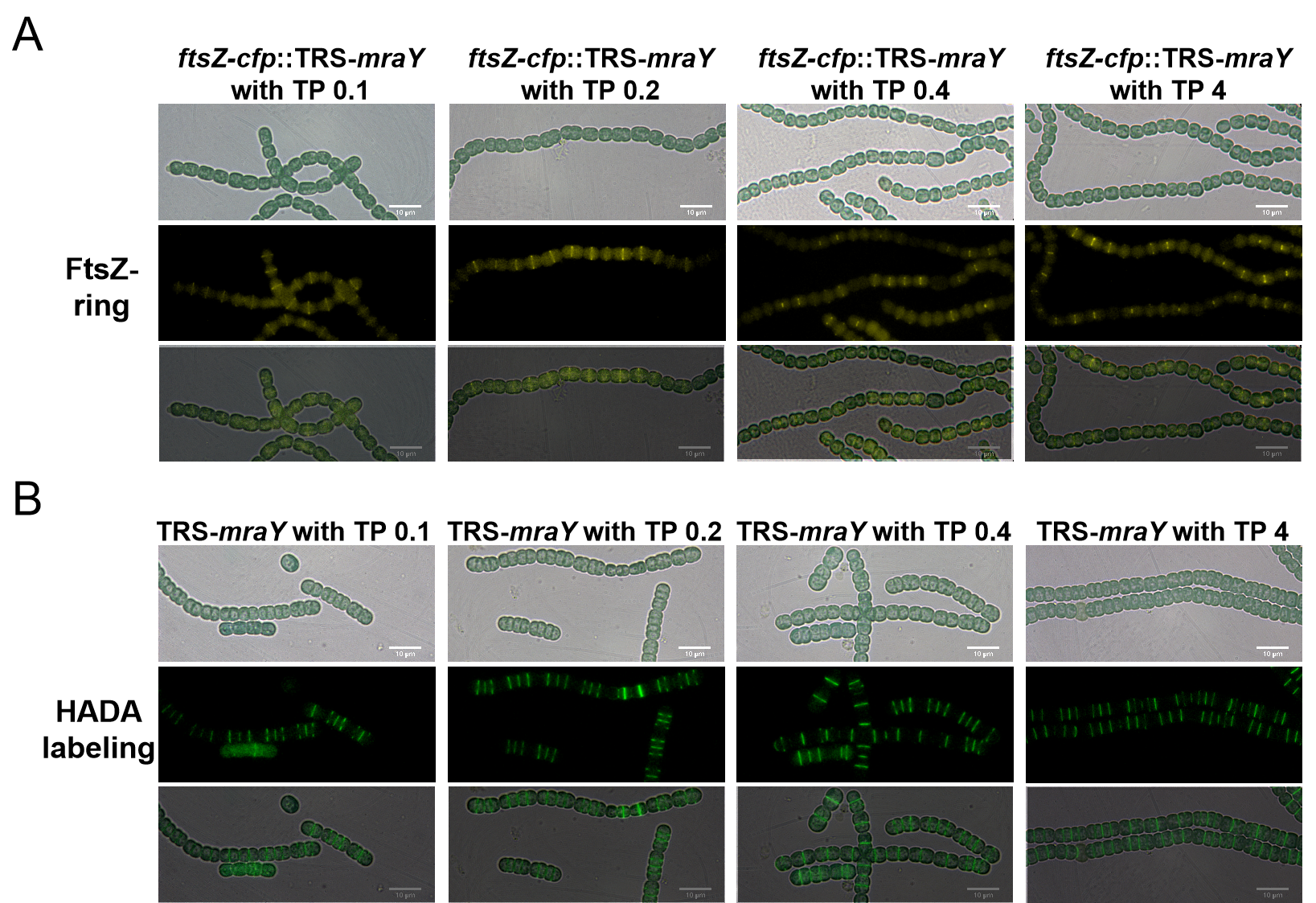
**

**Fig. S3:** FtsZ-ring formation and PG synthesis in TRS-*mraY* in BG11 with different concentrations of TP. (**A**) FtsZ-CFP is a CFP fluorescence fusion protein of FtsZ as already reported (Xing et al., 2021). Filaments of strain *ftsZ-cfp*::TRS-*mraY* grown in BG11 medium with 1 mM of TP were collected and transferred to BG11 medium without TP for 12 hours (Fig. S2) to remove TP. Then they were incubated for 24 hours in BG11 with different concentrations of TP to observe FtsZ localization. (**B**) Strain TRS-*mraY* was first treated similarly to remove TP (Fig. S2), then incubated for 24 hours with 150 µM of HADA in BG11 with different concentrations of TP to observe PG synthesis pattern. WT was used as the control.

**
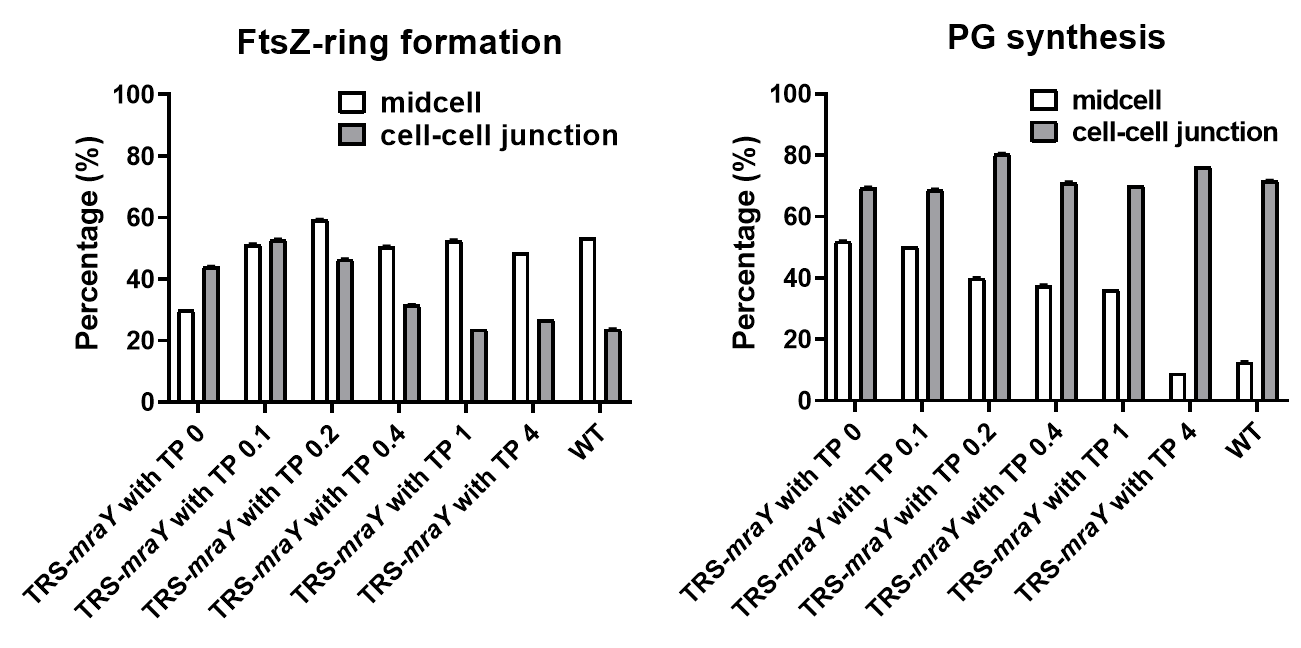
**

**Fig. S4:** FtsZ-ring formation and PG synthesis pattern in the wild type (WT) and strain TRS-*mraY*. The localization of FtsZ-CFP, or PG synthesis probed with HADA staining, was analyzed according to the data from Table 1.


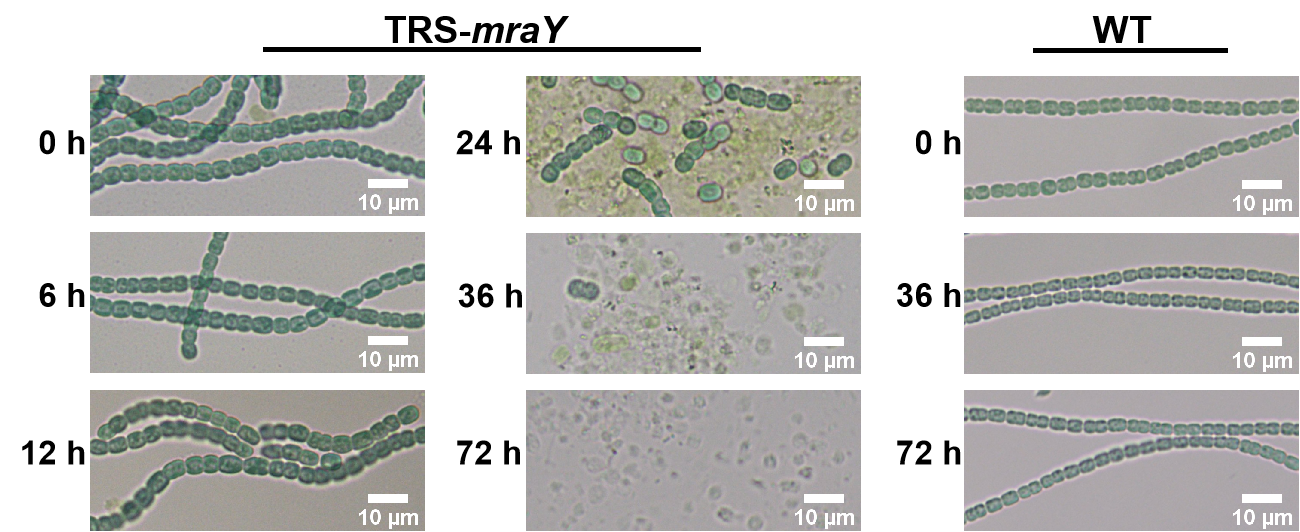


**Fig. S5:** The morphology of cells and filaments of strain TRS-*mraY* under non-permissive conditions. Filaments of strain TRS-*mraY* grown in BG11 medium with 1 mM of TP were collected and transferred to BG11 medium (without TP), in which they were incubated for the indicated number of hours under culture conditions. Samples of the cell suspensions were directly observed under a light microscope and photographed. WT was used as the control. The size in indicated by a bar.


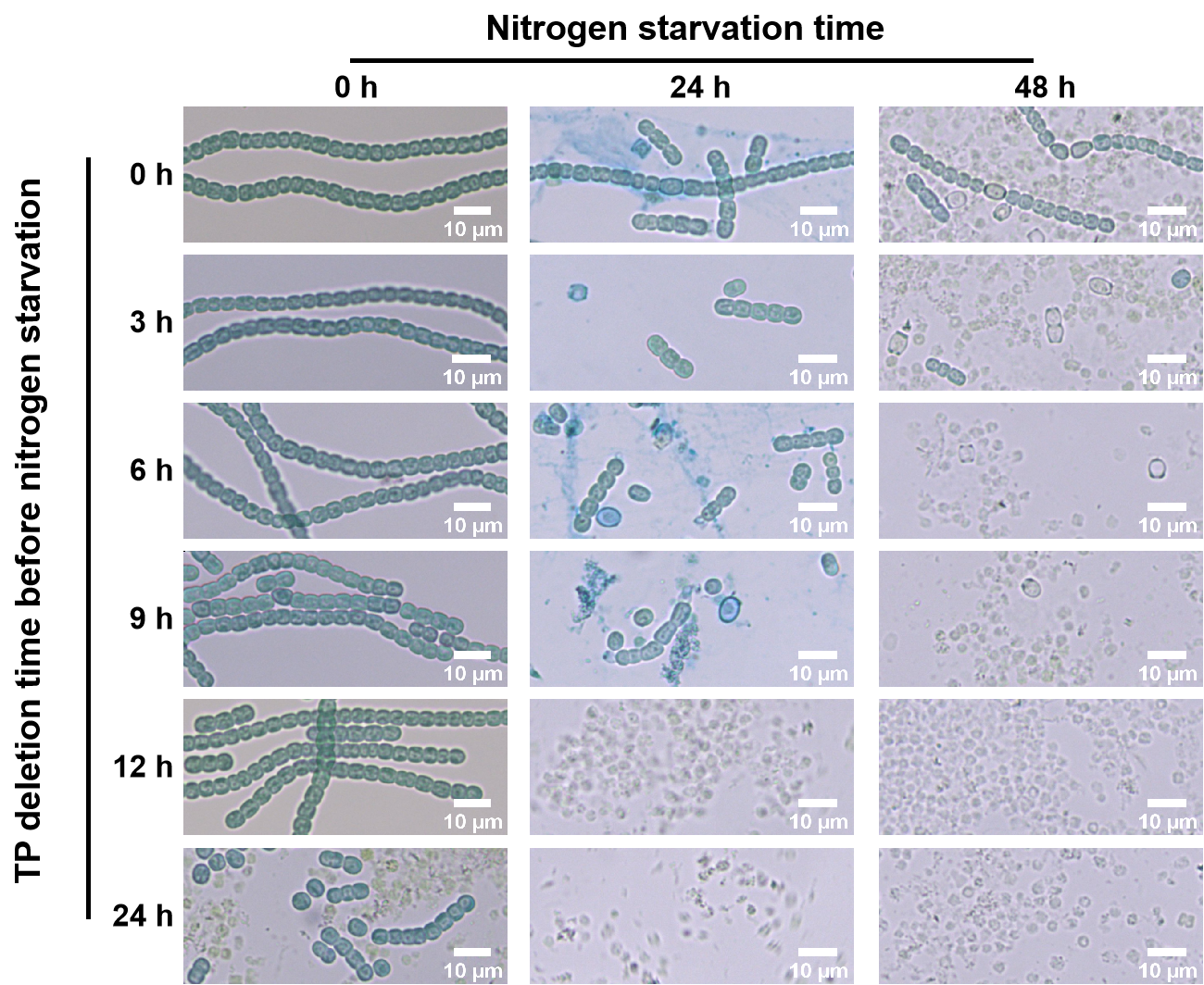


**Fig. S6:** Heterocyst differentiation of TRS-*mraY* in BG11_0_ after diffirent TP deletion time. Filaments of strain TRS-*mraY* grown in BG11 medium with 1 mM of TP were collected and transferred to BG11 medium without TP, in which they were pre-incubated for different TP deletion time under culture conditions before nitrogen starvation. Then filaments were collected and transferred to BG11_0_ medium without TP. To better visualize protereocysts or heterocysts, filaments were incubated with alcian blue which stains the polysaccharide layer of these cells. Samples of the cell suspensions were directly photographed after the indicated time of incubation after nitrogen stepdown. Magnification was the same for all micrographs.

**Fig. S3:** The heterocyst differentiation of TRS-*mraY* in BG11_0_ after diffirent TP deletion time. Filaments of strain TRS-*mraY* grown in BG11 medium with 1 mM of TP were collected and transferred to BG11 medium without TP, in which they were pre-incubated for different TP deletion time under culture conditions before nitrogen starvation. Then filaments were collected and transferred to BG11_0_ medium without TP. Samples of the cell suspensions were directly photographed after the indicated time of incubation. Magnifcation was the same for all micrographs.

**Fig. S4:** MraY is involved in filament integrity. Filament length of WT and TRS-*mraY* mutant with different concentrations of TP were analysed after 4 day culture. The number of cells per filament means the filament length, and filaments with 100 cells include those with more than 100 cells per filament. 300 filaments were counted for filament length.

**REFERENCES**

1. Niu TC, Lin GM, Xie LR, Wang ZQ, Xing WY, Zhang JY, Zhang CC. 2018. Expanding the potential of CRISPR-Cpf1 based genome editing technology in the cyanobacterium *Anabaena* PCC 7120. ACS Synth Biol 8:170-180. https://doi.org/10.1021/acssynbio.8b00437.

2. Xing WY, Xie LR, Zeng X, Yang Y, Zhang CC. 2020. Functional dissection of genes encoding DNA polymerases based on conditional mutants in the heterocyst-forming cyanobacterium *Anabaena* PCC 7120. Front Microbiol 11:1108. <https://doi.org/10.3389/fmicb.2020.01108>.

3. Battesti A, Bouveret E. 2012. The bacterial two-hybrid system based on adenylate cyclase reconstitution in *Escherichia coli*. Methods 58:325-334. https://doi.org/10.1016/j.ymeth.2012.07.018.

4. Xing WY, Liu J, Wang ZQ, Zhang, JY, Zeng XL, Yang YL, and Zhang CC. 2021. HetF protein is a new divisome component in a filamentous and developmental cyanobacterium. mBio 12: e01382-21. https://doi.org/10.1128/mBio

.01382-21.
